# Randomised clinical trial comparing concomitant and hybrid therapy for eradication of *Helicobacter pylori* infection

**DOI:** 10.1371/journal.pone.0244500

**Published:** 2020-12-30

**Authors:** Antonio Mestrovic, Nikola Perkovic, Josko Bozic, Mirela Pavicic Ivelja, Jonatan Vukovic, Goran Kardum, Zeljko Puljiz, Ante Tonkic

**Affiliations:** 1 Department of Gastroenterology, University Hospital of Split, Split, Croatia; 2 Department of Pathophysiology, University of Split School of Medicine, Split, Croatia; 3 Department for Infectious Diseases, University Hospital of Split, Split, Croatia; 4 Department of Psychology, Faculty of Humanities and Social Sciences, University of Split, Split, Croatia; 5 Department of Internal Medicine, University of Split School of Medicine, Split, Croatia; Hvidovre Hospital, DENMARK

## Abstract

**Background:**

The primary objective of this study was to compare concomitant and hybrid therapy in the first line eradication treatment of *Helicobacter pylori* infection in Split-Dalmatia County, Croatia, in which clarithromycin resistance is above 20%. The secondary objective of the study was to determine and compare compliance and adverse events rate between these therapeutic protocols.

**Materials and methods:**

In an open-label, randomised clinical trial 140 patients total with *H*. *pylori* infection were randomly assigned to either concomitant (esomeprazole 40 mg, amoxicillin 1 g, metronidazole 500 mg, clarithromycin 500 mg, twice daily for 14 days) or hybrid (esomeprazole 40 mg and amoxicillin 1 g twice daily during 14 days with adding metronidazole 500 mg and clarithromycin 500 mg twice daily, in the last 7 days,) treatment group.

**Results:**

Eradication rates for concomitant group and hybrid therapy group were 84.1% (58/69) and 83.1% (59/71) respectively in the intention-to-treat analysis and 96.7% (58/60) and 95.2% (59/62) in per-protocol analysis. There was no significant difference between the groups (ITT analysis: *P* = 0.878; PP analysis: *P* = 0.675). Adverse events were more frequent in the concomitant group (33.3% vs 18.3%, *P* = 0.043). There was no difference among groups regarding compliance rate.

**Conclusion:**

Hybrid therapy has similar eradication rate as concomitant therapy, with lower adverse events rate. In the era of increasing antibiotic resistance, eradication regime with less antibiotic’s usage, as hybrid therapy, should be reasonable first line treatment choice for *H*. *pylori* infection.

Clinical Trials, gov: NCT03572777.

## Introduction

Although introduced as a first-line carcinogen years ago, *H*. *pylori* is still a clinical challenge, due its association with gastritis, gastric and duodenal ulcer, MALT (mucosa-associated lymphoid tissue) and gastric cancer [1–4].

The 2015 Kyoto Consensus defined *H*. *pylori* gastritis as an infectious disease, requiring treatment regardless of symptomatology [5]. In this regard, the choice of appropriate eradication therapy is important, as eradication can prevent above mentioned complications [6, 7]. However, an increase in *H*. *pylori* resistance to antibiotics has been reported worldwide, with a concurrent decline in the success of eradication therapy, necessitating the need for a modification of the therapeutic approach [8, 9]. This is further supported by the fact that traditional triple therapy is no longer considered the therapy of choice in areas of high resistance to clarithromycin (>15%) [3].

Therefore, a model of quadruple therapy was proposed by the *H*. *pylori* Working Group (Maastricht V): sequential, concomitant, hybrid, and quadruple bismuth-based therapies. Hybrid therapy, proposed in 2011 by Hsu, is a combination of sequential and concomitant therapy [10]. Few clinical studies so far showed the same or even higher eradication rate of hybrid therapy compared to sequential and concomitant regimes [11, 12].

Considering the fact that there is no defined optimal eradication therapy for *H*. *pylori* infection that would be equally effective in all regions, it is advised to first determine primary resistance to commonly used antibiotics in eradication of *H*. *pylori* infection in each region [3, 13]. To our knowledge, the efficacy of hybrid therapy in the treatment of *H*. *pylori* in Croatia has not been investigated to date. Given that the choice of eradication therapy is primarily based on local antibiotic resistance, we consider it is essential to examine the efficacy of hybrid and concomitant therapy in *H*. *pylori* eradication in the Split-Dalmatia area, knowing that the clarithromycin resistance is above 20% in our region [14]. In this study we compared concomitant and hybrid therapy for *H*. *pylori* infections, in terms of efficacy, compliance and adverse events rate.

## Methods

### Design overview

We conducted a prospective, open-label, randomised, controlled trial at the University Hospital of Split in Croatia. Between July 2018 and August 2019, all patients who presented with dyspeptic symptoms or had endoscopic finding (peptic ulcer, gastritis) have been tested for *H*. *pylori* infection. Patients with *H*. *pylori* infection were initially recruited in study and were followed up until October 2019. *H*. *pylori* infection was proven with one of the following methods: positive stool antigen assay (based on monoclonal antibody, ELISA); positive rapid urease test; *H*. *pylori* evidence in histologic specimen; positive urea breath test, all in accordance with the recent Maastricht V guidelines. Exclusion criteria were: age less than 18 years; previously unsuccessful application of empirical *H*. *pylori* eradication therapy; malignant disease of the stomach or any other site; taking proton pump inhibitors (PPI), H2 antagonists, bismuths or antibiotics (amoxicillin, metronidazole, clarithromycin) during the last month; associated comorbidity (renal insufficiency, mental illness); drug allergies: proton pumps inhibitors or antibiotics (amoxicillin, metronidazole, clarithromycin); pregnancy and lactation; refusal to participate in the survey.

All participants provided written informed consent. The study was performed in accordance with the principles of good clinical practice from the Declaration of Helsinki, approved by the ethic committees of the University Hospital of Split (as from April 2018, approval number 500-03/18-01/13) and University of Split School of Medicine (as from April 2018; approval number: 003-08/18-03/0001) and registered as clinical trial (Clinical Trials, gov: NCT03572777). The authors confirm that all ongoing and related trials for this drug/intervention are registered.

Data regarding participant’s demographic and baseline characteristics (age, gender, endoscopic findings (gastric/duodenum ulcer, erosive gastritis erosive duodenitis), smoking data and alcohol consumption)) were collected.

### Therapy

The eligible participants were randomly assigned, using computer generating sequence in two groups. First group was given concomitant therapy: esomeprazole 40 mg, amoxicillin 1 g, clarithromycin 500 mg and metronidazole 500 mg, which were all administered orally twice daily for a total of 14 days. The second group was given hybrid therapy: esomeprazole 40 mg and amoxicillin 1 g, which were administered orally twice daily for a total of 14 days and clarithromycin 500 mg and metronidazole 500 mg, which were administered orally twice daily for the last seven days. Written instructions on the dose and timing of treatment were provided to each subject individually.

One month after the end of therapy, all subjects were tested for *H*. *pylori* antigen in the stool using a monoclonal antibody (ELISA) test to evaluate eradication success. Eradication failure was defined as a positive result of this test. During the follow-up, compliance and adverse events were evaluated. The compliance was defined by the amount of medications taken (compliance was considered good if ≥ 80% of therapy was taken), based on the remaining pill count and patient’s self-reported questionnaire that included information regarding compliance and adverse events.

The adverse events included: nausea, abdominal pain, diarrhoea, constipation, dizziness, metal taste (in mouth), headache, loss of appetite, vomiting, skin rash, itching, black tongue, tongue deposits.

The adverse events were divided into groups according to the degree of tolerance: no adverse events; mild (without limitation in daily activities); moderate (partly limited daily activities); and severe (completely limited daily activities). Patients were instructed to report immediately in case of any severe adverse events.

The primary outcome of the study was to compare *H*. *pylori* eradication rates in patients receiving concomitant and hybrid therapy. Secondary outcomes were assessment of compliance and adverse events in the both groups.

### Statistical analysis

The total number of participants was calculated based on the effect size parameter (w = 0.5), statistical significance (*P* = 0.01), and power of 0.90. Based on the input parameters, a sample size of 60 subjects per group was required. Sample size calculations was made using power analysis statistical package in the R interface (ver. 3.4.3, 2017).

Statistical software SPSS ver. 25 for Windows (IBM Corp, Armonk, NY, USA) was used for statistical data analysis. Data were expressed as mean ± standard deviation (SD) or as whole numbers and percentages with 95% confidence intervals (CIs) calculation for categorical variables. The major outcomes were analyzed by the Chi-squared test with Yates’ correction or Fisher’s exact test for categorical data and the Student’s t-test for continuous variables. Binomial logistic regression analysis, with age and gender variables as covariates, was used to determine adjusted odds ratios (aOR) for adverse events of the hybrid therapy group with concomitant therapy group set as a reference group. Analysis was performed by intention-to-treat (ITT) and per protocol (PP). The ITT population included all randomised patients who received at least one dose of study drugs. The PP analysis excluded the patients with unknown *H*. *pylori* status following therapy and patients with poor compliance to the therapy. All assumptions for the use of statistical tests have been fulfilled. The statistical significance was defined as *P* < 0.05.

## Results

### Study group characteristics

**Fig 1:**
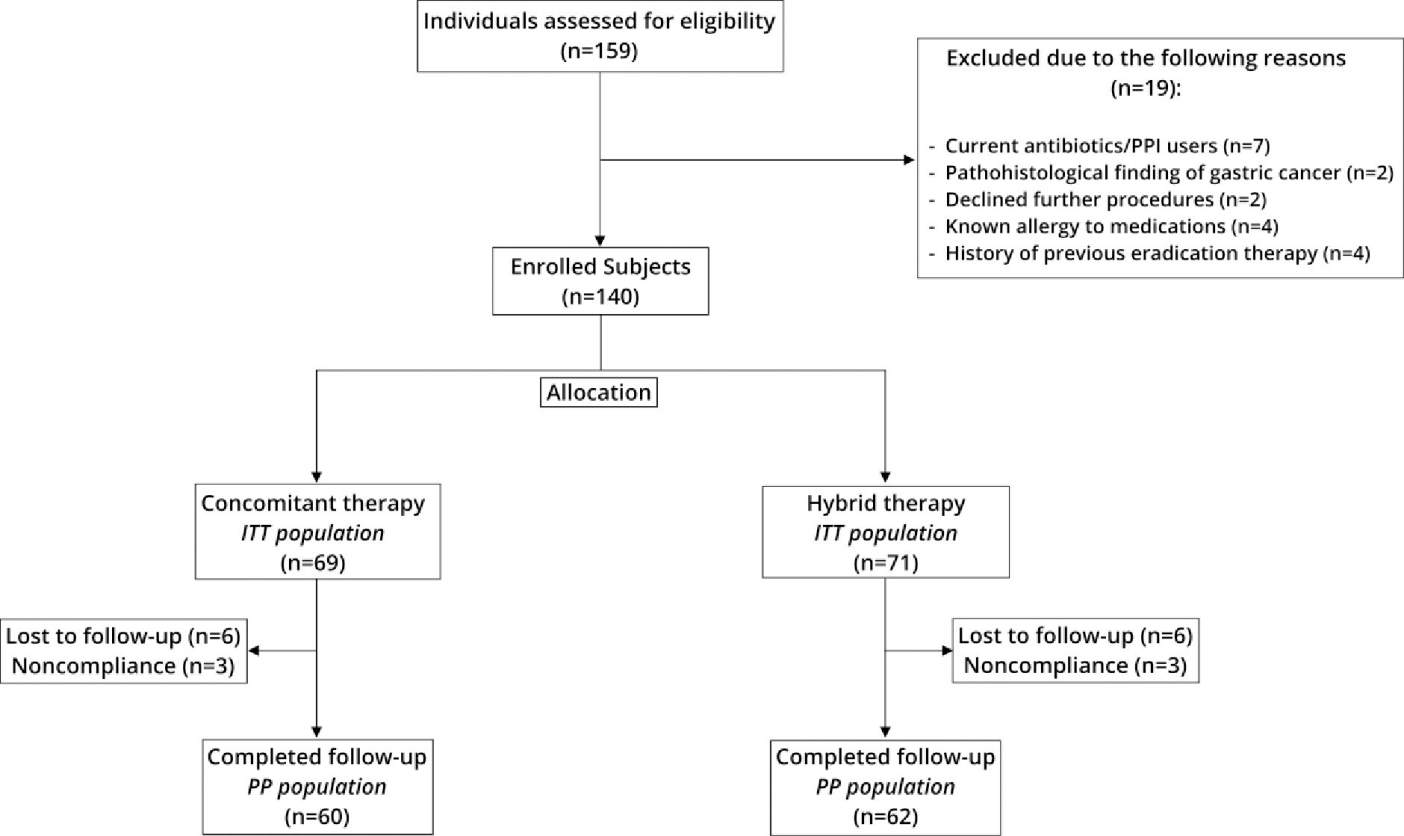
Study flowchart.

Among 159 patients infected with *H*. *pylori* 19 were excluded due to screening failure. A total of 140 patients were randomly assigned to either concomitant therapy (n = 69) or hybrid therapy (n = 71) group. Table 1 shows baseline characteristics of the included patients. There were no statistically significant differences between the two groups in terms of age, sex, history of smoking, alcohol use, or endoscopic finding. Six patients total in the concomitant group and six patients in hybrid group were lost to follow-up. In each group, three patients consumed less than 80% of prescribed medications. A flowchart of the recruitment of study participants is shown in the Fig 1.

**Table 1:** Baseline characteristics of study population.

Parameter	Concomitant therapy (N = 69)	Hybrid therapy (N = 71)	*P*
Male gender	37 (53.6)	44 (62.0)	0.319^a^
Age (years)	61.6 ± 13.1	59.5 ± 12.1	0.317^b^
Smoking	17 (24.6)	17 (23.9)	0.924^a^
Alcohol consumption	12 (17.4)	14 (19.7)	0.724^a^
Endoscopic findings			
Gastritis	53 (76.8)	56 (78.8)	0.983^c^
Gastric ulcer	10 (14.5)	10 (14.1)	
Duodenal ulcer	5 (7.2)	4 (5.6)	
Duodenitis	1 (1.4)	1 (1.4)	

Data are presented as whole number and percentage or mean ± standard deviation

^a^ Chi-squared test

^b^ t-test for independent samples

^c^ Fisher's exact test

### Outcomes

For the intention-to-treat (ITT) analysis, the eradication rates of *H*. *pylori* were 84.1% (58/69; 95% CI: 77.0–91.2) in the concomitant group and 83.1% (59/71; 95% CI: 75.8–90.4) in the hybrid therapy group (*P* = 0.878). For the per-protocol (PP) analysis, the eradication rates were 96.7% (58/60; 95% CI: 93.4–100.0) in the concomitant group and 95.2% (59/62; 95% CI: 91.0–99.4) in the hybrid group (*P* = 0.675). There were no significant differences in the eradication rate between the two groups, according to the ITT and PP analyses (Table 2).

**Table 2:** Clinical outcomes of study population.

Parameter	Concomitant therapy (N = 69)	Hybrid therapy (N = 71)	*P*^*a*^
Eradication rate			
Intention-to-treat N (%; 95% CI)	58/69 (84.1; 77.0–91.2)	59/71 (83.1; 75.8–90.4)	0.878
Per-protocol N (%; 95% CI)	58/60 (96.7; 93.4–100.0)	59/62 (95.2; 91.0–99.4)	0.675
Compliance >80%	60/69 (87.0)	62/71 (87.3)	0.986
Adverse event	23/69 (33.3)	13/71 (18.3)	0.043

Data are presented as whole number and percentage

^a^ Chi-squared test

### Compliance and adverse events

There was no significant difference in the compliance rate between the two groups. Nine patients in both concomitant and hybrid group had compliance rate below 80%.

**Fig 2:**
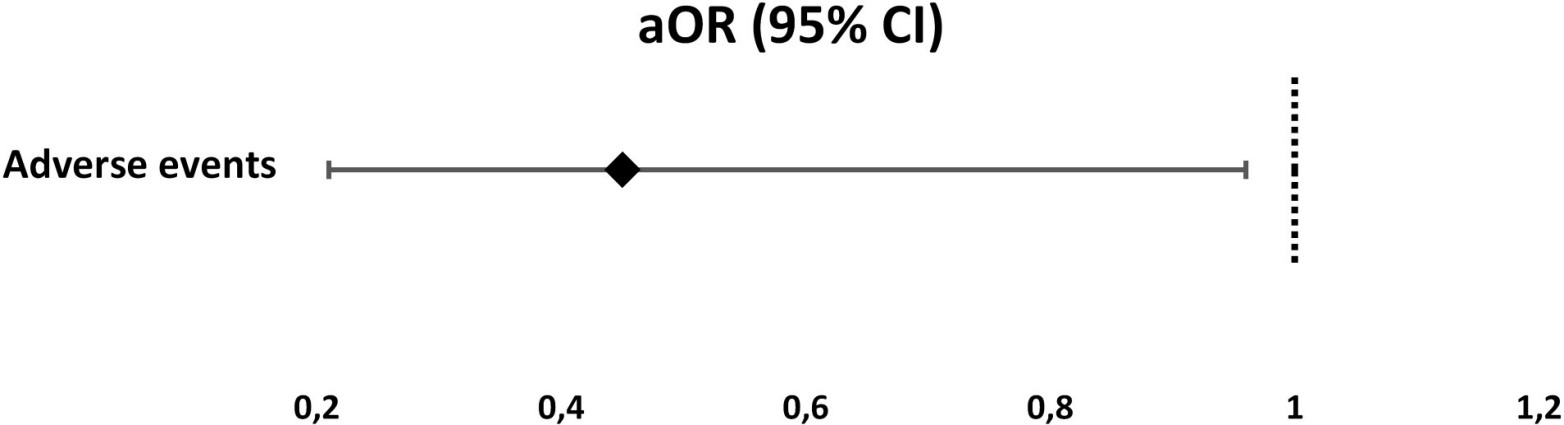
Adjusted odds of adverse events in the hybrid therapy group. ^a^Reference group is *Concomitant therapy* group.

Adverse events occurred significantly higher in concomitant than in hybrid group (33.3% vs 18.3%, *P* = 0.043). Furthermore, hybrid group had significantly lower adjusted odds of adverse events (aOR 0.45, 95%CI 0.21–0.96, *P* = 0.044) as shown in Fig 2. Nausea was the most frequent adverse event in both groups (20.3% and 11.3% respectively), as it is shown in the Table 3. According to the degree of severity, most of the adverse events were mild in both groups (19/69 in concomitant and 13/71 in hybrid group). However, four patients in the concomitant therapy group experienced moderate adverse events, but without need for special intervention or hospitalization (Table 4).

**Table 3:** Analysis of all adverse events of the study population^c^.

Parameter	Concomitant therapy (N = 69)	Hybrid therapy (N = 71)	*P*
Nausea	14 (20.3)	8 (11.3)	0.142^a^
Abdominal pain	2 (2.9)	0 (0.0)	-
Diarrhoea	2 (2.9)	1 (1.4)	0.543^b^
Constipation	1 (1.4)	0 (0.0)	-
Dizziness	1 (1.4)	1 (1.4)	0.984^b^
Metallic taste	2 (2.9)	3 (4.2)	0.672^b^
Headache	1 (1.4)	0 (0.0)	-
Loss of appetite	0 (0.0)	1 (1.4)	-
Vomiting	1 (1.4)	1 (1.4)	0.984^b^
Skin rash	2 (2.9)	1 (1.4)	0.543^b^
Pruritus	0 (0.0)	1 (1.4)	-
Black tongue	0 (0.0)	1 (1.4)	-
Tongue deposits	2 (2.9)	1 (1.4)	0.543^b^

Data are presented as whole number and percentage

^a^ Chi-squared test

^b^ Fisher's exact test

^c^ Some patients had more than one adverse effect

**Table 4:** Analysis of adverse event severity.

Adverse event ^b^	Concomitant therapy (N = 69)	Hybrid therapy (N = 71)	*P*^a^
None	46 (66.7)	58 (81.7)	0.028
Mild	19 (27.5)	13 (18.3)	
Moderate	4 (5.8)	0 (0)	

Data are presented as whole number and percentage

^a^ Fisher's exact test

^b^ Severe adverse events have not been reported.

## Discussion

The primary objective of this study was to determine the optimal therapeutic option in the treatment of *H*. *pylori* infection since it is not clearly defined in Split-Dalmatia region, Croatia. According to previously established data, clarithromycin resistance in Split-Dalmatia County is above 20%, with relatively low metronidazole resistance rate of 10.2% [14]. Therefore, standard triple therapy is not recommended as a first line treatment [3]. As stated in Maastricht V guidelines, in areas with high (>15%) clarithromycin resistance, bismuth quadruple or non-bismuth quadruple therapies, primarily concomitant, are recommended [3]. Concomitant therapy is now often regarded as the first line eradication treatment, due its high eradication rate, exceeding 90% in some areas [15–17].

However, standard duration of concomitant therapy is from 10 to 14 days, that includes PPI and three antibiotics–amoxicillin, metronidazole, clarithromycin, which are used for total period of treatment. This can lead to increase of antibiotic resistance and abuse of antibiotic use. Furthermore, as suggested by Maastricht and Toronto guidelines, concomitant therapy is duration dependent, with preferable 14-day duration in the first attempt, especially in areas with high clarithromycin resistance [4, 13]. On the other hand, results of Kapizioni et al. study suggested that 10-day concomitant therapy could replace 14-day therapy with equal result [18]. Secondly, significant limitation of concomitant therapy can be lower efficacy in areas with high dual resistance or high metronidazole resistance, when bismuth-based therapy is recommended [19, 20]. Results of one meta-analysis demonstrated that eradication rate of concomitant therapy was only 33.3–66.7% for strains with dual clarithromycin-metronidazole resistance [21].

To overcome these problems, other quadruple therapies, such as sequential and hybrid were proposed. Hybrid therapy was introduced as a novel non-bismuth quadruple therapy in 2011, with excellent first results: eradication rate was 99.1% by PP and 97.4% by ITT analysis [10]. So far, the effectiveness of hybrid therapy since its introduction has been investigated in few studies, and there are even less studies with comparison of concomitant and hybrid therapy [22–27].

Meanwhile, sequential therapy, first introduced as an alternative to triple therapy, was a common first line treatment in Croatia [27]. Soon, few studies showed that hybrid can be more effective than sequential therapy [12, 28]. However, usage of sequential therapy showed limitations. In areas with high clarithromycin resistance sequential therapy can be less effective than concomitant therapy [29]. Efficacy of sequential therapy drops down significantly when *H*. *pylori* strains were clarithromycin‐resistant, even down to 70%, as presented by Liou et al. [30]. There is also evidence that sequential therapy is affected by metronidazole resistance [3].

On the other hand, hybrid therapy showed better eradication rate than sequential therapy in areas with high antibiotic resistance, as showed in Sardarian et al. study [28]. Thus, in our region, we have chosen hybrid therapy as an alternative option to concomitant therapy.

In the current study we have demonstrated similarly high eradication rates for concomitant and hybrid therapy and these findings are consistent with results of few other studies in areas with high clarithromycin resistance [24–26, 28, 31, 32].

Given the fact that the therapy is time-dependent, we have chosen 14 days duration for both therapy groups, similarly to other authors [29, 32]. This is in contrary with previous studies that used 10-day hybrid, or 10-day concomitant therapy [24, 26, 33]. However, one prospective Greek study showed high eradication rate using 14-day hybrid therapy, in a region with similar antibiotic resistance rate which was the main reason for us to use 14-day therapy [25, 34]. Hybrid therapy includes 7 days less taking metronidazole and clarithromycin, but with equal eradication success. It seems that hybrid therapy would be more reasonable approach, having in mind increasing antibiotic abuse and antibiotic resistance. This is strengthened by the fact that eradication of *H*. *pylori* can be associated with changes in gut microbial ecology and structure [35, 36]. In addition, hybrid therapy is more cost-effective than the concomitant therapy. When we compare costs of both therapies, hybrid first line treatment is less expensive, primarily because of 7-day shorter antibiotic (metronidazole and clarithromycin) usage.

The secondary objectives of the study were to determine the tolerability of these therapeutic protocols and to evaluate patients’ quality of life during treatment based on adverse events occurrence.

In all therapeutic regimes, compliance rate could be another potential factor for eventual failure of eradication treatment. In our study, in both groups compliance rate was more than satisfactory, with no significant difference, although we expected better compliance in hybrid group, regarding a smaller number of antibiotics, as some studies showed [24, 31].

As we expected, less antibiotic usage resulted in less adverse events. We demonstrated significantly higher adverse events rate in concomitant than in hybrid group, with nausea being the most common adverse event in both groups. There were no differences in specific adverse events among groups. Adverse events were mild according to the degree of severity, and four patients who had moderate events were in concomitant group. Furthermore, hybrid group had significantly lower adjusted odds of adverse events.

Similar finding was in one study, with less adverse events in hybrid group and nausea being dominant complaint [24]. Few other studies proved there was no difference regarding adverse events [22, 26, 31, 32].

Although, this is the first randomised clinical trial comparing hybrid and concomitant therapy in Croatia, our study has few limitations. Main limitation is lack of antibiotic resistance data for included patients, however, current guidelines recommend antibiotic susceptibility test after second line treatment failure [3]. Still, tailored therapy in the era of personalized medicine should be regarded as a potential future approach in clinical practice. Secondly, this study was designed as open-label one, which may increase potential risk of bias. Although majority of similar *H*. *pylori* clinical trials are open-label, blind-design studies are necessary for avoiding potential bias [24, 25, 27, 35]. Finally, this study was not designed as non-inferiority one, that may affect its conclusiveness. Thus, a non-inferiority trial should be conduct for further comparison of these two protocols, with greater sample size.

We used 14-day concomitant and 14-day hybrid regime. However, further studies need to be performed to investigate the potential benefit of 10-day hybrid regime in both eradication efficacy and compliance. In that manner, potential risk of increase in antibiotic resistance would be even more avoided. Regarding the fact that our region has clarithromycin resistance rate above 20%, results of this study may be applicable with regions with similar problem.

In conclusion, both concomitant and hybrid therapy achieved very high but similar eradication rates. The scientific contribution of this clinical research is clarifying efficacy of therapeutic protocols (ITT> 90%) in the treatment of *H*. *pylori* infection in patients in Split-Dalmatia County. Regarding the lesser number of antibiotics, less adverse events and similar eradication rate, we suggest that hybrid therapy should be the first line treatment option in areas with high clarithromycin resistance. Further studies are needed to investigate potential usage of 10-day hybrid therapy compared with 14-day regime.

## Supporting information

S1 Appendix Consort checklist. (DOC)

S2 Appendix Study protocol English. (DOCX)

S3 Appendix Study protocol Croatian. (DOCX)
