# Supplementary material for: Randomised clinical trial comparing concomitant and hybrid therapy for eradication of Helicobacter pylori infection
Source: PLoS One. 2020 Dec 30;15(12):e0244500. doi: 10.1371/journal.pone.0244500 (PMC7773256; doi:10.1371/journal.pone.0244500)
Supplement: S2 Appendix — (DOCX) [file pone.0244500.s002.docx]

**University Hospital of Split, Split, Croatia**

**Department of Internal Medicine**

**Department of Gastroenterology**

**Antonio Meštrović, MD**

**Phone: +385 098/9123462**

**e-mail: antonio.mestrovic1@gmail.com**

**Ethic committee**

**University Hospital of Split**

**Spinčićeva 1, 21000 Split**

**Croatia**

**Ethic committee**

**University of Split School of Medicine**

**Šoltanska 2, 21000 Split**

**Croatia**

**Study title:** A randomized controlled study: quadruple vs hybrid therapy in the treatment of *Helicobacter pylori* infection

**Basic data and study protocol**

Although introduced as a first-line carcinogen several years ago, *Helicobacter pylori* is still a clinical challenge. *H. plyori* infection has been associated with gastritis, gastric and duodenal ulcer, MALT (mucosa-associated lymphoid tissue), and gastric cancer (1). The 2015 Kyoto Consensus defined *H. pylori* gastritis as an infectious disease, requiring treatment, regardless of symptomatology (2). In this regard, the choice of appropriate eradication therapy is important, as eradication leads to the cure of gastritis, which can prevent the complications above. However, an increase in *H. pylori* resistance has been reported worldwide, with a decline in the success of eradication therapy, necessitating the need of modification of therapeutic approach. This is further supported by the fact that traditional triple therapy is no longer considered the therapy of choice in areas of high resistance (> 15%) to clarithromycin (3,4). Therefore, a model of quadruple therapy was proposed by the *Helicobacter pylori* Working Group (Maastricht V): sequential, concomitant, hybrid, and quadruple bismuth-based therapies, with the duration of therapy to 14 days (3,5). Hybrid therapy, proposed in 2011 by the *Helicobacter pylori* working group, is a combination of sequential and concomitant therapy (6). Few clinical studies so far demonstrate the same efficacy of hybrid therapy compared to sequential and concomitant one (3,7).

According to recent guidelines (Maastricht V) the optimal eradication therapy for *Helicobacter pylori* infection which would be equally effective in all regions, it in to defined. It is advised to determine primary antibiotic resistance to *Helicobacter pylori* prior of postulating eradication regime guidelinnes in the region. To our knowledge, the effectiveness of hybrid therapy in the treatment of *H. pylori* in Croatia has not been examined to date. The choice of eradication therapy should primarily be based on data of local antibiotic resistance in order to optimize the selection of suitable eradication therapy (8-10).

**Primary objective of the study:**

1. To compare the efficacy of concomitant and hybrid therapy in the treatment of *Helicobacter pylori* infection.

**Secondary objectives**:

2. Determine patients's compliance in concomitant and hybrid therapeutic groups.

3. Determine adverse events in concomitant and hybrid therapy groups.

4. To determine the quality of life of patients with *Helicobacter pylori* infection before and after eradication therapy using the Gastrointestinal Symptom Rating Scale (GSRS) scale.

5. Compare the quality of life of patients before and after taking eradication therapy between groups treated with hybrid and concomitant therapy in the treatment of *Helicobacter pylori* infection.

**Hypothesis:**

1. The efficacy of concomitant therapy in the treatment of *Helicobacter pylori* infection is above 90%.

2. The effcacy of hybrid therapy in the treatment of *Helicobacter pylori* infection is above 90%.

3. Patients treated with hybrid therapy have better compliance than patients treated with concomitant therapy.

4. Patients treated with hybrid therapy have fewer side effects than patients treated with concomitant therapy.

5. The quality of life of patients after successful eradication therapy is better than in patients in whom eradication of *Helicobacter pylori* infection has not been successful.

**Expected scientific contribution of the research**

The primary objective of this study is to determine the optimal therapeutic option in the treatment of *Helicobacter pylori* infection since it is not clearly defined in Croatia. Based on recent consensus, today there is no optimal eradication therapy that would be appropriate in all European countries and in the world. Based on current guidelines, modern *Helicobacter pylori* infection eradication therapy should be based on local / regional monitoring of primary antibiotic resistance to commonly used antibiotics in eradication therapy, as well as clinical evaluation of the efficacy of therapeutic protocols based primarily on data on local clarithromycin resistance. The primary objective of this trial is to determine which therapeutic option (in accordance with the recommendations of the recent Maastricht Conference) would be optimal in the treatment of *Helicobacter pylori* infection in Split-Dalmatia County, given the previously established data regarding the antibiotic resistance. It should be noted that data on primary resistance to commonly used antibiotics in eradication of *Helicobacter pylori* infection exist only for the Split-Dalmatia County (in which clarithromycin resistance is above 20%) (8,9).

The secondary objectives of the study would be to determine the tolerability of these therapeutic protocols and to evaluate the quality of life of the patient during treatment (compliance and side effects).

The scientific contribution of this clinical research is to determine the effectiveness of therapeutic protocols (ITT> 90%) in the treatment of *Helicobacter pylori* infection in patients in Split-Dalmatia County, which is not clearly defined today.

**Type of study**: an open-label, randomized clinical trial.

**Duration of the research:**

The estimated duration of the study is from April 15, 2018 to October 15, 2019.

**Information about the study site:**

The study will be conducted at the Department of Gastroenterology and Hepatology, Department of Internal Medicine, and at the Deparment of Microbiology and Parasitology, University Hospital of Split, Split, Croatia.

**Number of participants:**

Total number of patients: 140 (expected)

Number of patients in hybrid therapy group: 70 (ITT)

Number of patients in concomitant therapy group: 70 (ITT)

**Inclusion Criteria:**

- *Helicobacter pylori* infection (documented by: positive antigen stool test, or positive urease rapid test obtained during endoscopy, or *Helicobacter pylori* in histological finding, or positive C-urea breath test; according to recent Maastricht V guidelines;
- written informed consent

**Exclusion Criteria:**

- age under 18
- previously treatment of *Helicobacter pylori* infection
- malignancy of stomach or other site
- history of taking proton pump inhibitors, H2 antagonist, bismuth or antibiotics (amoxicillin, metronidazole, clarithromycin) in previous month
- significant underlying disease (renal failure, psychiatric disorders)
- history of allergic reactions to any medications used in study
- refuse to participate in study
- breastfeeding and pregnancy
- quitting the study

**Statistical analysis**

The primary analysis of this study focuses on the eradication rate of *Helicobacter pylori* in two groups, those taking concomitant and those receiving hybrid therapy. Based on previous research, an eradication rate greater than 90% is expected in both the concomitant and hybrid therapy groups. The results of the analysis will use the chi-square test and, where appropriate, the Fisher exact test and Yates correction, and possibly the proportionality test. Statistical significance will be weighed at 95% (*P* <0.05). The total number of patients using the chi-square test was calculated based on the effect size parameter (w = 0.3), statistical significance (*P* = 0.05), and power of 0.90. Based on the input parameters, a total sample size of n = 117 is required. In the case of a proportionality test and a difference of 5% between concomitant and hybrid therapy, the sample size should be slightly larger (N = 174) with an effect size of 0.30 and a power of 0.80. Sample size calculations were made using pwr and powerAnalysis statistical packages in the R interface (ver. 3.4.3, 2017).

**Research Protocol:**

The study will include all outpatients and / or inpatients at the Department of Gastroenterology with *H. pylori* infections proved with: positive stool immunochromatographic test; positive urease rapid test; evidence of *Helicobacter pylori* in a histological specimen taken during EGDS; positive urea breathing test. All patients must have signed informed consent and research procedure will be explained to them. Patients will be randomized into two groups.

Demographic and anamnestic characteristics (age, gender, endoscopic findings (gastric / duodenal ulcer, (erosive) gastritis, erosive duodenitis), smoking data (quantity and duration) and alcohol consumption (quantity and duration)) will be recorded in all patients. The GSRS (Gastrointestinal Symptom Rating Scale) questionnaire, which is specific to the evaluation of gastrointestinal disorders, will be distributed to the patients and will take up to 5 minutes to complete, consisting of 15 questions divided into five groups of disorders: reflux disorders, abdominal pain, maldigestion, diarrhea, constipation. The patients gives one of seven possible answers to each question (in grading system: 1 without interference, 7 very severe interference; according to the Likert scale).

Concomitant therapy will then be administered to one group: esomperazole 40 mg 2x1 tablets, amoxicillin 1 gr 2x1 tablets, clarithromycin 500 mg 2x1 tablets and metronidazole 500 mg 2x1 tablets for a total of 14 days. The second group will be administered hybrid therapy: esomperazole 40 mg 2x1 tablets and amoxilicin 1 gram 2x1 tablets for a total of 14 days, with the addition of clarithromycin 500 mg 2x1 tablets and metronidazole 500 mg 2x1 tablets for the last seven days. Written instructions on the dose and timing of treatment will be given to each patient individually.

One month after the end of therapy, all patients will be tested for *Helicobacter pylori* antigen in the stool using a monoclonal antibody (ELISA) at the Deparment of Microbiology. The findings will be provided during a control examination, when all patients will be be asked regarded compliance and possible adverse events while taking therapy.

Compliance will be defined by the amount of medication taken (good compliance will be considered to be ≥ 80% of therapy taken), which the patient will prove by showing the rest of the drug.

We will divide the adverse events into groups according to the degree of severity: no side effects; mild side effects (without limitations of daily activities); moderate (partly limited daily activities); heavy (completely limited daily activities).

At the same time, to all patietns will again be given a GSRS questionnaire, which they will need to complete.

Finally, we will statistically analyze the success of eradication of *Helicobacter pylori* infection in both groups (concomitant and hybrid) separately and compare the result. We will compare patient compliance as well as adverse events.

We will also compare the quality of life, based on the results of the GSRS questionnaire, of the patients before and after eradication therapy.

References:

1. Fock KM, Graham DY, Malfertheiner P. Helicobacter pylori research: historical insights and future directions. Nat Rev Gastroenterol Hepatol 2013;10:495–500.

2. Sugano K, Tack J, Kuipres E J, et al. Kyoto global consensus report on Helicobacter pylori gastritis[J]. Gut 2015;64(9):1353-1367.

3. Malfertheiner P, Megraud F, O'Morain CA et al. Management of Helicobacter pylori infection-the Maastricht V/Florence Consensus Report. European Helicobacter and Microbiota Study Group and Consensus panel. Gut 2017 Jan;66(1):6-30. doi: 10.1136/gutjnl-2016-312288. Epub 2016 Oct 5.

4. Megraud F, Coenen S, Versporten A, et al. Helicobacter pylori resistance to antibiotics in Europe and its relationship to antibiotic consumption. Gut 2013;62:34-42.

5. Fallone CA, Chiba N, van Zanten SV et al. The Toronto Consensus for the Treatment of Helicobacter pylori Infection in Adults. Gastroenterology 2016;151(1):51-69.e14.

6. HsuPI, Wu DC, Wu JY, Graham DY. Modified sequential Helicobacter pylori therapy: proton pump inhibitor and amoxicillin for 14 days with claritromycin and metronidazole added as a quadruple (hybrid) therapy for the final 7 days. Helicobacter 2011;16:139-145.

7. Heo J, Jeon SW, Jung JT et al. Concomitant and hybrid therapy for Helicobacter pylori infection: A randomized clinical trial. J Gastroenterol Hepatol 2015;30:1361–1366. doi:10.1111/jgh.12983

8. Tonkic A, Tonkic M, Brnic D, Novak A, Puljiz Z, Simunic M. Time trends of primary antibiotic resistance of Helicobacter pylori isolates in Southern Croatia. J Chemother. 2012 Jun;24(3):182-4.

9. Tonkić A, Tonkić M, Brnić D. Increasing prevalence of primary clarithromycin resistance in Helicobacter pylori strains in Split, Croatia. J Chemother. 2009 Nov;21(5):598-9.

10. Tonkić M, Tonkić A, Goić-Barisić I, Jukić I, Simunić M, Punda-Polić V. Primary resistance and antibiotic minimum inhibitory concentrations for Helicobacter pylori strains, in Split, Croatia. J Chemother. 2006 Aug;18(4):437-9.

11. Lee HJ, Kim JI, Lee JS, et al. Concomitant therapy achieved the best eradication rate for Helicobacter pylori among various treatment strategies. World Journal of Gastroenterology 2015;21(1):351-359.

12. Song Z-Q, Zhou L-Y. Hybrid, sequential and concomitant therapies for Helicobacter pylori eradication: A systematic review and meta-analysis. World Journal of Gastroenterology 2016;22(19):4766-4775.

Principle investigator: Antonio Meštrović, MD

Signature: ___________
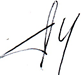
_____________

In Split 8th March, 2018

Study protocol

Enrolled patients

N =

uključeni ispitanici

n=

uključeni ispitanici

n=

| Excluded due to criteria |
| --- |

allocation

Hybrid therapy

N = (ITT)

Concomitant therapy

N = (ITT)

Noncompliance

N =

Noncompliance

N =

Lost to follow up

N =

Lost to follow-up

N =

Concomitant therapy

N = (PP)

Hybrid therapy

N = (PP)
