# Supplementary material for: Randomised clinical trial comparing concomitant and hybrid therapy for eradication of Helicobacter pylori infection
Source: PLoS One. 2020 Dec 30;15(12):e0244500. doi: 10.1371/journal.pone.0244500 (PMC7773256; doi:10.1371/journal.pone.0244500)
Supplement: S3 Appendix — (DOCX) [file pone.0244500.s003.docx]

**KLINIČKI BOLNIČKI CENTAR SPLIT**

**Klinika za unutarnje bolesti**

**Zavod za gastroenterologiju i hepatologiju**

Antonio Meštrović, dr.med.

tel: 098/9123462

e-mail: antonio.mestrovic1@gmail.com

**Etičko povjerenstvo**

**Klinički bolnički centar Split**

**Spinčićeva 1, 21000 Split**

**Naziv istraživanja:** **Randomizirano kliničko istraživanje: usporedba četverostruke konkomitantne i hibridne terapije u liječenju *Helicobacter pylori* infekcije**

A randomized controlled study: quadruple vs hybrid therapy in the treatment of *Helicobacter pylori* infection

**Osnovni podatci i plan istraživanja**

Iako je prije više godina predstavljen kao karcinogen prvog reda, *Helicobacter pylori* još uvijek predstavlja klinički izazov. Infekcija *Helicobacter plyori* povezuje se s nastankom gastritisa, želučanog i duodenualnog ulkusa, MALT (mucosa-associated lymphoid tissue) limfoma te karcinoma želuca (1). Kyoto konsenzus iz 2015. godine definirao je *Helicobacter pylori* gastritis kao infektivnu bolest, koja zahtijeva liječenje, bez obzira na simptomatologiju (2). U tom pogledu, važan je izbor prikladne eradikacijske terapije, obzirom da eradikacija dovodi do izliječenja gastritisa, što može prevenirati gore navedene komplikacije. Međutim, porast rezistencije *H. pylori* bilježi se u čitavom svijetu, uz istodobni pad uspješnosti eradikacijske terapije, što nalaže potrebu modifikacije terapijskog pristupa. Tome u prilogu govori i činjenica da se tradicionalna trojna terapija, više ne smatra terapijom izbora u područjima visoke rezistencije (>15%) na klaritromicin (3,4). Stoga je od strane *Helicobacter pylori* radne skupine (Maastricht V), predložen model četverostruke terapije: sekvencijske, konkomitantne, hibridne te četverostruke terapije temeljene na bizmutu, dok se samo trajanje terapije produljuje na 14 dana (3,5). Hibridna terapija, predložena 2011. godine od strane *Helicobacter pylori* radne skupine, predstavlja spoj sekvencijske i konkomintante terapije (6). Dosadašnja malobrojna klinička istraživanja pokazuju podjednaku učinkovitost hibridne terapije u usporedbi sa sekvencijskom i konkomitantnom terapijom (3,7).

Obzirom da, temeljem važećih konsenzus konferencija (Maastricht V) nije definirana optimalna eradikacijska terapija za *Helicobacter pylori* infekciju koja bi bila jednako učinkovita u svim regijama, savjetovano je da se u svakoj regiji determinira prethodno određivanje primarne rezistencije na najčešće korištene antibiotike u eradikaciji *Helicobacter pylori* infekcije kao i evaluaciji terapijskih protokola koji su postulirani u Maastricht V smjernicama. Koliko nam je poznato, do sada nije ispitivana učinkovitost hibridne terapije u liječenju *H. pylori* u Hrvatskoj. Obzirom da je izbor eradikacijske terapije prvenstveno temeljen na lokalnoj antibiotskoj rezistenciji (8-10), smatramo esencijalnim ispitati učinkovitost hibridne i konkomitantne terapije u eradikaciji *H. plyori* na splitsko-dalmatinskom području, kako bi što više optimizirali izbor prikladne eradikacijske terapije.

**Cilj istraživanja:**

1. Usporediti učinkovitost četverostruke konkomitantne i hibridne terapije u liječenju *Helicobacter pylori* infekcije.

Sekundarni ciljevi:

1. Utvrditi suradljivost bolesnika tijekom uzimanja konkomitantne i hibridne terapije u liječenju *Helicobacter pylori* infekcije.
2. Utvrditi moguće nuspojave tijekom uzimanja konkomitantne i hibridne terapije u liječenju *Helicobacter pylori* infekcije.
3. Utvrditi kvalitetu životu bolesnika s *Helicobacter pylori* infekcijom prije i nakon eradikacijske terapije uz pomoć *Gastrointestinal Symptom Rating Scale* (GSRS) ljestvice.
4. Usporediti kvalitetu života bolesnika prije i nakon uzimanja eradikacijske terapije između skupina bolesnika kod kojih je primijenjena hibridna te konkomitantna terapija u liječenju *Helicobacter pylori* infekcije.

**Hipoteza:**

1. Učinkovitost četverostruke konkomitantne terapije u liječenju *Helicobacter pylori* infekcije je iznad 90 %.
2. Učinkovitost hibridne terapije u liječenju *Helicobacter pylori* infekcije je iznad 90 %.
3. Bolesnici liječeni hibridnom terapijom imaju veću suradljivost tijekom liječenja, nego bolesnici liječeni četverostrukom konkomitantnom terapijom.
4. Bolesnici liječeni hibridnom terapijom imaju manje nuspojava tijekom liječenja, nego bolesnici liječeni četverostrukom konkomitantnom terapijom.
5. Kvaliteta života bolesnika nakon uspješne eradikacijske terapije bolja je nego kod bolesnika kod kojih eradikacija *Helicobacter pylori* infekcije nije bila uspješna.

**Očekivani znanstveni doprinos istraživanja:**

Primarni cilj ovog istraživanja je utvrđivanje optimalne terapijske opcije u liječenju *Helicobacter pylori* infekcije obzirom da ona u Hrvatskoj nije jasno definirana. Temeljem recentnih konsenzusa danas ne postoji optimalna eradikacijska terapija koja bi bila primjerena u svim europskim zemljama pa tako ni u cijelom svijetu. Temeljem važećih smjernica moderna terapija eradikacije *Helicobacter pylori* infekcije treba se zasnivati na lokalnom/regionalnom praćenju primarne antibiotske rezistencije na najčešće korištene antibiotike u eradikacijskoj terapiji kao i na kliničkoj evaulaciji učinkovitosti terapijskih protokola, temeljenih prvenstveno na podatku o lokalnoj rezistenciji na klaritromicin (4). Upravo je i primarni cilj ovog ispitivanja utvrditi koja bi terapijska opcija (sukladno preporukama recentne Maastricht konferencije) bila optimalna u liječenju *Helicobacter pylori* infekcije u Splitsko-dalmatinskoj županiji, a obzirom na prethodno utvrđene podatke. Za napomenuti je da podatci o primarnoj rezistenciji na najčešće korištene anbitiotike u eradikaciji *Helicobacter pylori* infekcije postoje samo za Splitsko-dalmatinsku županiju (u kojoj je rezistencija na klaritromicin iznad 20 %) (8,9).

Sekundarni ciljevi istraživanja bili bi utvrđivanje suradljivosti bolesnika te eventualnih nuspojava te ocjena kvalitete života bolesnika tijekom uzimanja terapije.

Znanstveni doprinos kliničkog istraživanja očituje se u utvrđivanju učinkovitosti terapijskih protokola (>90 %) u liječenju *Helicobacter pylori* infekcije u pacijenata u Splitsko-dalmatinskoj županiji koja danas, kao ni u cijeloj zemlji, nije jasno definirana.

**Vrsta istraživanja:**

Prospektivno, otvoreno, randomizirano kliničko istraživanje.

**Trajanje istraživanja:**

Predviđeno trajanje istraživanje je od 15. travnja 2018. do 15. listopada 2018. godine.

**Podaci o mjestu provođenja istraživanja:**

Istraživanje će se provoditi u Zavodu za gastroenterologiju i hepatologiju Klinike za unutarnje bolesti te u Kliničkom zavodu za mikrobiologiju i parazitologiju Kliničkog bolničkog centra Split.

**Broj ispitanika:**

Ukupni broj ispitanika: 140 (očekivani).

Broj ispitanika liječen hibridnom terapijom: 70 (ITT).

Broj ispitanika liječen konkomitantnom terapijom: 70 (ITT).

**Kriteriji uključivanja:**

1. bolesnici s dokazanom *Helicobacter pylori* infekcijom (jednom od sljedeće navedenih metoda: pozitivan imunokromatografski test iz stolice; pozitivan ureaza brzi test prilikom ezofagogastroduodenoskopije (EGDS); dokaz *Helicobacter pylori* u histološkom uzorku uzetom tijekom EGDS-a; pozitivan ureja izdisajni test), a sukladno posljednjim, Maastricht V smjernicama;

2. potpisani informirani pristanak na sudjelovanje u istraživanju.

**Kriteriji isključivanja:**

1. dob bolesnika manja od 18 godina;

2. prethodno neuspješna primjena empirijske *Helicobacter pylori* eradikacijske terapije;

3. maligna bolest želuca ili drugog sijela;

4. uzimanje inhibitora protonske pumpe, H2 antagonista, bizmuta ili antibiotika (amoksicilin, metronidazol, klaritromicin) u posljednih mjesec dana;

5. pridruženi komorbiditet (renalna insuficijencija, psihička bolest);

6. odbijanje sudjelovanja u istraživanju;

7. podatak o alergijama na lijekove: inhibitor protonske pumpe ili antibiotike (amoksicilin, metronidazol, klaritromicin);

8. trudnoća i dojenje;

9. odustanak ispitanika od sudjelovanja u istraživanju.

**Statistika:**

Primarna analiza ovog rada usmjerena je na eradikacijski udio *Helicobacter pylori* u dvije skupine, oni koji uzimaju konkomitantnu i oni koji primaju hibridnu terapiju. Temeljem prethodnih istraživanja, i u konkomitantnoj i u hibridnoj terapijskoj skupini očekuje se eradikacijski udio veći od 90%. U analizi rezultata koristit će se hi kvadrat test i po potrebi Fisher egzaktni test i Yates korektura te moguće i test proporcija. Statistička značajnost će se odmjeriti na razini od 95% (p<0.05). Ukupan broj ispitanika pri korištenju hi kvadrat testa računa se temeljem parametra veličine učinka (w=0.3), statističke značajnosti (p=0.05) i snage istraživanja od 0.90. Na osnovi ulaznih parametara potrebna je ukupna veličina uzorka od n=117. U slučaju korištenja testa proporcije i razlike od 5% između konkomitantne i hibridne terapije, veličina uzorka bi trebala biti nešto veća i iznosti n=174 uz veličinu učinka od 0.30 i snage od 0.80. Izračun veličine uzorka napravljen je pomoću statističkih paketa pwr i powerAnalysis u R sučelju (ver. 3.4.3, 2017).

**Protokol istraživanja:**

Istraživanjem će biti obuhvaćeni svi ambulantni i/ili bolnički bolesnici u Zavodu za gastroenterologiju i hepatologiju kod kojih se dokaže *Helicobacter pylori* infekcije na jedan od navedenih načina: pozitivan imunokromatografski test iz stolice; pozitivan ureaza brzi test prilikom ezofagogastroduodenoskopije (EGDS); dokaz *Helicobacter pylori* u histološkom uzorku uzetom tijekom EGDS-a; pozitivan ureja izdisajni test. Svi ispitanici moraju imati potpisani infomirani pristanak, a prethodno će im se objasniti postupak istraživanja. Ispitanici će biti randomizirani u dvije skupine, računalnom metodom.

Kod svih ispitanika će biti evidentirane demografske i anamnestičke karakteristike (dob, spol, endoskopski nalaz (ulkus želuca/duodenuma, (erozivni) gastritis, erozivni duodenitis), podatak o pušenju (količina i trajanje) i konzumaciji alkohola (količina i trajanje)). Nakon toga ispitanicima će biti podijeljen GSRS (Gastrointestinal Symptom Rating Scale) upitnik, specifičan za evaluaciju gastrointestinalnih smetnji, za čije je ispunjavanje potrebno do 5 minuta, a sastoji se od 15 pitanja podijeljenih u pet skupina smetnji: refluksne smetnje, bol u trbuhu, maldigestija, proljev, konstipacija. Na svako pitanje ispitanik daje jedan od mogućih sedam odgovora (po sustavu gradiranja: 1 bez smetnji, 7 vrlo teške smetnje; sukladno Likertovoj ljestvici).

Potom će jednoj skupini biti ordinirana četverostruka konkomitantna terapija: esomeprazol 40 mg 2x1 tableta, amoksicilin a 1 gr 2x1 tableta, klaritromicin a 500 mg 2x1 tableta te metronidazol a 500 mg 2x1 tableta, u trajanju od ukupno 14 dana. Drugoj skupini će biti ordinirana hibridna terapija: esomeprazol a 40 mg 2x1 tableta te amoksilicin 1 gram 2x1 tableta u trajanju od ukupno 14 dana, uz dodatak klaritromicina a 500 mg 2x1 tableta te metronidazola a 500 mg 2x1 tableta posljednjih sedam dana. Pisane upute o dozi i vremenu uzimanja terapije bit će dane svakom ispitaniku pojedinačno.

Mjesec dana nakon završetka terapije kod svih ispitanika bit će proveden test antigena *Helicobacter pylori* u stolici služeći se monoklonskim protutijelom (ELISA), u Kliničkom zavodu za mikrobiologiju i parazitologiju. S nalazom će se javiti na kontrolni pregled, kada će kod svih ispitanika biti provedeno ispitivanje, o suradljivosti i eventualnim nuspojavama tijekom uzimanja terapije.

Suradljivost će biti definirana količnom uzetih lijekova (dobrom suradljivosti smatrat će se ≥ 80 % uzete terapije), što će ispitanik dokazati donošenjem ostatka lijeka.

Nuspojave ćemo podijeliti u skupine s obzirom na stupanj podnošenja: bez nuspojava; blage nuspojave (bez ograničenja na dnevne aktivnosti); umjerene (dijelom ograničene dnevne aktivnosti); teške (potpuno ograničene dnevne aktivnosti).

Ujedno će ponovno svim ispitanicima biti dan GSRS upitnik, kojeg će morati ispuniti.

Na kraju ćemo statistički analizirati uspješnost eradikacije *Helicobacter pylori* infekcije u obje skupine (konkomitantna i hibridna) zasebno te usporediti rezultat. Usporedit ćemo suradljivost bolesnika među skupinama kao i moguće nuspojave. Također ćemo usporediti kvalitetu života, temeljem rezultata GSRS upitnika, ispitanika prije i nakon eradikacijske terapije.

**Reference:**

1. Fock KM, Graham DY, Malfertheiner P. Helicobacter pylori research: historical insights and future directions. Nat Rev Gastroenterol Hepatol 2013;10:495–500.
2. Sugano K, Tack J, Kuipres E J, et al. Kyoto global consensus report on Helicobacter pylori gastritis[J]. Gut 2015;64(9):1353-1367.
3. Malfertheiner P, Megraud F, O'Morain CA et al. Management of Helicobacter pylori infection-the Maastricht V/Florence Consensus Report. European Helicobacter and Microbiota Study Group and Consensus panel. Gut 2017 Jan;66(1):6-30. doi: 10.1136/gutjnl-2016-312288. Epub 2016 Oct 5.
4. Megraud F, Coenen S, Versporten A, et al. Helicobacter pylori resistance to antibiotics in Europe and its relationship to antibiotic consumption. Gut 2013;62:34-42.
5. Fallone CA, Chiba N, van Zanten SV et al. The Toronto Consensus for the Treatment of Helicobacter pylori Infection in Adults. Gastroenterology 2016;151(1):51-69.e14.
6. HsuPI, Wu DC, Wu JY, Graham DY. Modified sequential Helicobacter pylori therapy: proton pump inhibitor and amoxicillin for 14 days with claritromycin and metronidazole added as a quadruple (hybrid) therapy for the final 7 days. Helicobacter 2011;16:139-145.
7. Heo J, Jeon SW, Jung JT et al. Concomitant and hybrid therapy for Helicobacter pylori infection: A randomized clinical trial. J Gastroenterol Hepatol 2015;30:1361–1366. doi:10.1111/jgh.12983
8. Tonkic A, Tonkic M, Brnic D, Novak A, Puljiz Z, Simunic M. Time trends of primary antibiotic resistance of Helicobacter pylori isolates in Southern Croatia. J Chemother. 2012 Jun;24(3):182-4.
9. Tonkić A, Tonkić M, Brnić D. Increasing prevalence of primary clarithromycin resistance in Helicobacter pylori strains in Split, Croatia. J Chemother. 2009 Nov;21(5):598-9.
10. Tonkić M, Tonkić A, Goić-Barisić I, Jukić I, Simunić M, Punda-Polić V. Primary resistance and antibiotic minimum inhibitory concentrations for Helicobacter pylori strains, in Split, Croatia. J Chemother. 2006 Aug;18(4):437-9.
11. Lee HJ, Kim JI, Lee JS, et al. Concomitant therapy achieved the best eradication rate for Helicobacter pylori among various treatment strategies. World Journal of Gastroenterology 2015;21(1):351-359.
12. Song Z-Q, Zhou L-Y. Hybrid, sequential and concomitant therapies for Helicobacter pylori eradication: A systematic review and meta-analysis. World Journal of Gastroenterology 2016;22(19):4766-4775.

Glavni istraživač: Antonio Meštrović, dr.med.

Potpis______
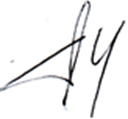
__________________

U Splitu, dana_09. ožujka 2018. godine

Protokol istraživanja

uključeni ispitanici

N =

uključeni ispitanici

n=

uključeni ispitanici

n=

randomizacija

konkomitantna terapija (ITT)

N =

hibridna terapija (ITT)

N =

loša suradljivost

N =

loša suradljivost

N =

odustali zbog nuspojava

N =

odustali zbog nuspojava

N =

nisu se javili na

kontrolni pregled

N =

nisu se javili na kontrolni pregled

N =

konkomitantna terapija (PP)

N =

hibridna terapija (PP)

N =
